# Predominantly Independent Genetic Control Between Growth and Visceral White Nodules Disease Resistance Revealed by High-Density Linkage Map and QTL Mapping in *Larimichthys crocea*

**DOI:** 10.3390/ijms27062531

**Published:** 2026-03-10

**Authors:** Ting Ye, Dandan Guo, Yilian Zhou, Bao Lou, Feng Liu

**Affiliations:** 1 State Key Laboratory for Managing Biotic and Chemical Threats to the Quality and Safety of Agro-Products, Institute of Hydrobiology, Zhejiang Academy of Agricultural Sciences, Hangzhou 310021, China; 15tye@stu.edu.cn (T.Y.); guodd@zaas.ac.cn (D.G.); zhouyl@zaas.ac.cn (Y.Z.); 2 Zhejiang Key Laboratory of Coastal Biological Germplasm Resources Conservation and Utilization, Wenzhou 325005, China

**Keywords:** *Larimichthys crocea*, high-density genetic map, QTL mapping, growth-disease resistance trade-off, genetic independence

## Abstract

The large yellow croaker (*Larimichthys crocea*) is a key mariculture species in China, however, its industry is threatened by visceral white nodules disease (VWND) caused by the bacterium *Pseudomonas plecoglossicida*. A significant challenge in breeding is the potential genetic trade-off between growth and disease resistance. To investigate their genetic relationship, we constructed a high-density SNP-based genetic linkage map for *L. crocea* using a F1 full-sib family (*n* = 150). The map comprised 24 linkage groups with 32,429 bin markers and an average interval of 0.051 cM. Based on this map, we conducted QTL mapping for one yield trait (body weight), eight morphological traits, and three VWND-resistance traits (survival time, AT; spleen and liver pathogen loads). Phenotypic analysis revealed strong integration among growth traits and a moderate positive correlation between growth traits and AT. QTL mapping identified 53 QTLs for growth (PVE = 0.14–5.83%) and 20 for resistance (PVE = 0.78–8.93%). Notably, only two genomic intervals exhibited co-localization between a morphological trait (AL or BL) and AT, each explaining a modest phenotypic variance (0.66–5.99%). The largest-effect QTLs for growth and resistance were mapped to distinct linkage groups, and candidate genes within the co-localized intervals (*Unc5d*, *SCN5A*, *HUS1*) are involved in fundamental cellular processes rather than core growth or immune pathways. These results suggest that yield, morphological, and VWND-resistance traits in *L. crocea* are largely under independent genetic control within the studied family, indicating that simultaneous improvement of growth and disease resistance is feasible. This study provides a molecular basis for breeding strategies aimed at overcoming the trait trade-off bottleneck in this economically vital species.

## 1. Introduction

The large yellow croaker (*Larimichthys crocea*) is a core mariculture fish species along the southeast coast of China, with an annual output of 257,000 tons nationwide in 2022, occupying an important position in the aquatic product industry [1]. In intensive aquaculture systems, visceral white nodules disease (VWND), caused by *Pseudomonas plecoglossicida*, poses a prominent threat, with a mortality rate of up to over 80%, resulting in substantial economic losses to major producing areas [2]. Unfortunately, pharmacological treatments have limited efficacy once infection occurs. Consequently, breeding disease-resistant strains has emerged as a safer and more sustainable alternative. Growth performance (including yield trait and morphological traits) and disease resistance are core breeding traits in *L. crocea*, and their genetic interaction (antagonism or synergy) is a critical issue for aquatic breeders. This contradiction is pervasive across animal and plant breeding, with extensive studies confirming the common existence of genetic trade-offs or synergies between growth performance and bacterial disease resistance. In aquatic animals, such trait interactions have been widely documented, reflecting inherent evolutionary and physiological constraints in genetic improvement [3]. Similarly, crop breeding research has consistently reported genetic interplay between growth and resistance, often manifesting as trade-offs that impede simultaneous enhancement of both traits [4,5]. The trade-off between these two traits has become an industrial bottleneck: selective breeding of disease-resistant strains alone tends to cause growth retardation, whereas excessive pursuit of rapid growth reduces population disease resistance. Clarifying the genetic correlation mechanism between them is thus the core premise for breaking this bottleneck.

Conventional approaches for genetic correlation analysis in aquatic animals mainly involve morphological phenotypic selection, family selection, population phenotypic correlation analysis, and quantitative genetic evaluation [6,7,8]. While effective for species with short generation cycles and high environmental controllability, these methods are particularly challenging for *L. crocea* due to its long generation cycle (2~3 years), high breeding costs, and phenotype plasticity in response to environmental factors such as temperature and diet. Consequently, traditional methods are not only protracted and costly but may also yield biased estimates of genetic correlations due to unresolved genotype-by-environment interactions, hindering the accurate localization of causative loci [9,10]. The advent of molecular markers has enabled more direct genetic analyses. Genome-wide association study (GWAS) has become a predominant tool in *L. crocea* research, largely circumventing the need for controlled familial construction [11,12,13]. However, robust GWAS requires large and diverse populations, and its correlative nature can struggle to distinguish between tight linkage and pleiotropy—a critical distinction for understanding the genetic relationship between complex traits like growth and disease resistance [1,14]. Linkage analysis based on high-density genetic mapping provides a complementary and powerful strategy for dissecting such trait correlations in structured pedigrees. It can accurately determine the genetic patterns (antagonism, synergy, or independence) between traits through locus overlap, linkage, or segregation, and has been widely applied in aquatic breeding [15,16,17].

Significant progress has been made in constructing genetic maps for *L. crocea*, laying a solid foundation for subsequent genetic research. However, inherent technical limitations of earlier studies have constrained in-depth investigations into trait correlations. Early AFLP maps developed by Ning et al. [18] and microsatellite maps by Ye et al. [19], with an average interval of 5.4 cM, provided valuable insights for preliminary QTL mapping of growth traits, though their relatively low marker density precluded fine-scale analysis of genetic interactions. The first SNP map constructed by Ao et al. [20] represented a notable advancement, reducing the average interval to 0.54 cM, yet it still faced challenges of marker segregation distortion and incomplete genome coverage. Furthermore, existing studies have focused on single traits either solely analyzing growth [11,19] or targeting a single disease [21,22] without investigating the correlation between growth and visceral white spot disease resistance, resulting in a lack of molecular basis for breeding practice.

Targeting the core breeding challenge of genetic interaction between growth performance and VWND-resistance, this study aimed to (1) construct a high-density SNP-based genetic linkage map for *L. crocea* using a full-sib F1 family and whole-genome resequencing, (2) perform comprehensive QTL mapping for one yield trait, eight morphological traits, and three VWND-resistance traits, and (3) elucidate the genetic relationship between growth and disease resistance through QTL co-localization and trait correlation analysis. It should be noted that the use of a single F1 family for QTL detection is a well-established approach in aquaculture genetic studies, as demonstrated in various species including *Luciobarbus brachycephalus* [17], *L. crocea* [22], and *Larimichthys polyactis* [23]. While multi-family designs can capture broader genetic diversity, single-family designs offer the advantage of controlled genetic background and reduced environmental noise, enabling precise QTL localization. In this study, although five families were initially generated, only one family retained an adequate sample size for robust QTL analysis due to the inherent challenges of long-generation-cycle aquaculture species, including environmental stressors during extended grow-out periods. Therefore, this study represents an initial step toward understanding the genetic architecture of growth and disease resistance in *L. crocea*, providing a foundation for future validation across broader genetic backgrounds.

## 2. Results

### 2.1. Phenotypic Variation and Normality Analysis of Growth and VWND Resistance Traits

**Figure 1:**
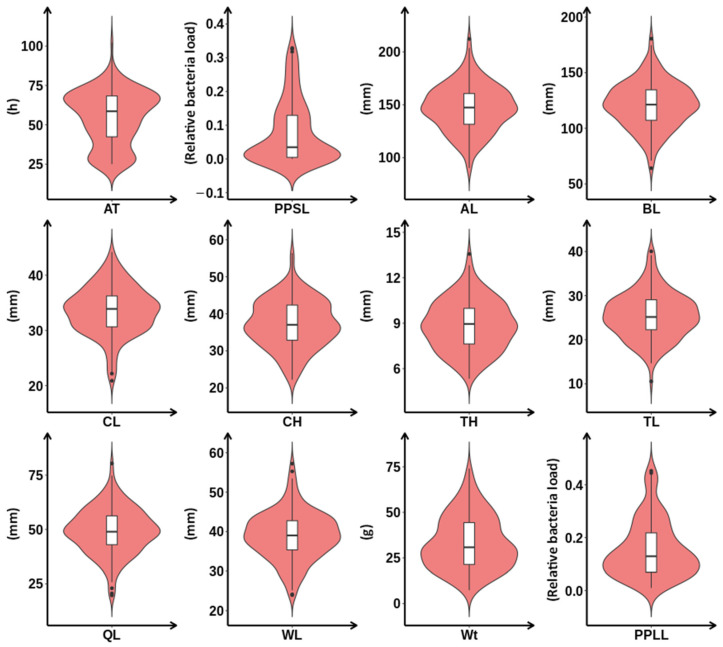
Frequency distribution of phenotypic values for the yield trait, eight morphological traits, and three VWND-resistance traits in the F1 mapping population of *L. crocea* (*n* = 150). The *x*-axis represents the phenotypic value for each trait, and the *y*-axis indicates the frequency of individuals. Black dots denote outlier samples. AL: total length; BL: body length; CH: body height; CL: head length; TH: tail height; TL: trunk length; QL: caudal peduncle length; WL: caudal fin length; Wt: body weight; AT: survival time; PPLL: relative abundance of *P. plecoglossicida* in liver; PPSL: relative abundance of *P. plecoglossicida* in spleen.

Phenotypic determination was performed on 150 F1 individuals of *L. crocea*, including 9 growth performance traits, yield trait (Wt), morphological traits (AL, BL, CH, CL, TH, TL, QL, WL), and 3 VWND resistance-related traits (AT, PPLL, PPSL). The phenotypic distribution characteristics and normality test results of all traits are shown in Figure 1 and Table S1, respectively. Figure 1 shows the frequency distribution of phenotypic values of all 12 traits. It can be observed that most morphological traits (AL, BL, CH, CL, TH, TL, WL) and one resistance trait (AT) exhibited an approximate normal distribution, with concentrated phenotypic values and uniform distribution trends. In contrast, the yield trait (Wt) and the other two pathogen load traits (PPLL, PPSL) displayed slight skewness, with phenotypic values skewed to the low-value region, indicating that most individuals had relatively low yield potential, liver pathogen load, and spleen pathogen load.

The normality test results (Table S1) further confirmed the distribution characteristics of the phenotypic data. The skewness values of AL, BL, CH, CL, TH, TL, WL, and AT ranged from −0.30 to 0.19, and the kurtosis values ranged from −0.80 to 0.28, all close to 0, indicating that these traits conformed to a normal distribution (*p* > 0.05). For Wt, PPLL, and PPSL, the skewness values were 0.49, 0.97, and 1.11, respectively, and the kurtosis values were −0.48, 0.28, and 0.16, respectively, showing mild to moderate positive skewness, which might be related to the genetic variation of these traits. Overall, all phenotypic data showed good genetic variation, with the coefficient of variation ranging from 8.92% (TH) to 87.50% (PPSL), providing a solid foundation for subsequent genetic correlation analysis and QTL mapping.

### 2.2. Phenotypic Correlation Among Yield, Morphological and VWND Resistance Traits

**Figure 2:**
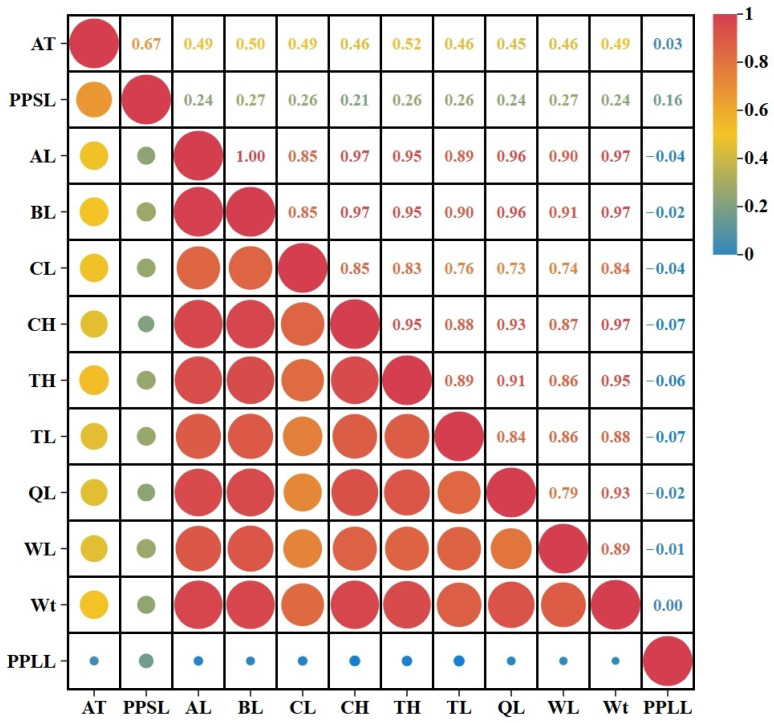
Heatmap of the Pearson correlation coefficients among the growth and VWND-resistance traits in *L. crocea* (*n* = 150). Correlation strength and direction are indicated by color intensity and hue: red denotes positive correlations, blue denotes weak or no correlation. Larger circles denote higher correlation coefficients.

Pearson correlation analysis was performed to assess the relationships among one yield trait, eight morphological traits, and three disease resistance traits in 150 individuals (Figure 2). A strong, significant positive correlation (*p* < 0.01) was observed among all morphological traits and between the yield trait (Wt) and each morphological trait, with correlation coefficients (r) ranging from 0.73 to 0.99 (Figure 2). Morphological traits AL and BL showed the highest correlation (r = 0.99). The yield trait Wt was highly correlated with BL and AL (r = 0.97), suggesting potential shared genetic regulation of growth and morphology. Among the resistance traits, survival time after challenge (AT) was significantly positively correlated with spleen pathogen load (PPSL) (r = 0.67, *p* < 0.01). In contrast, liver pathogen load (PPLL) showed only a weak, non-significant correlation with PPSL (r = 0.16) and no correlation with AT (r = 0.03), indicating distinct regulatory pathways for different resistance components.

Notably, both the yield trait (Wt) and all morphological traits showed positive correlations with survival trait AT (r = 0.45–0.52, *p* < 0.01) and showed weak positive correlations with PPSL (r = 0.21–0.27, *p* < 0.05). However, no significant correlations were detected between yield/morphological traits and hepatic pathogen load (PPLL) (r = −0.07–0.03, *p* > 0.05). These results indicate that individuals with superior growth and morphology exhibited longer survival and moderately higher splenic pathogen burden following challenge, whereas hepatic pathogen load was independent of growth and morphological phenotype.

### 2.3. Linkage Analysis and Genetic Map Construction

**Figure 3:**
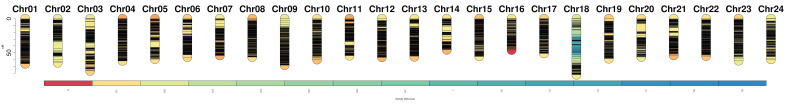
High-density consensus genetic linkage map of *L. crocea* constructed using the pseudo-testcross strategy. The map comprises 24 linkage groups (LGs) corresponding to the haploid chromosome number of the species.

A high-resolution genetic linkage map was constructed for *L. crocea* based on whole-genome resequencing of a full-sib F1 family, following the pseudo-testcross strategy and using filtered bin markers. The resulting consensus map consisted of 24 linkage groups (LGs) (Figure 3) corresponding to the 24 chromosomes of the species (Table 1). A total of 32,429 bin markers were integrated, spanning a total genetic length of 1417.17 cM, with an average inter-marker distance of 0.051 cM. The linkage groups ranged in genetic length from 45.72 cM (LG14) to 82.43 cM (LG18), and the number of mapped markers per LG varied from 377 (LG18) to 1862 (LG4). The average marker interval across LGs ranged from 0.033 cM (LG1, LG4, LG15, LG16) to 0.219 cm (LG18), indicating high marker density and uniform distribution. More than 99.5% of adjacent marker pairs were separated by less than 1 cM, confirming minimal gaps and comprehensive genome coverage.

#### Quality Assessment and Collinearity of the Genetic Map

**Figure 4:**
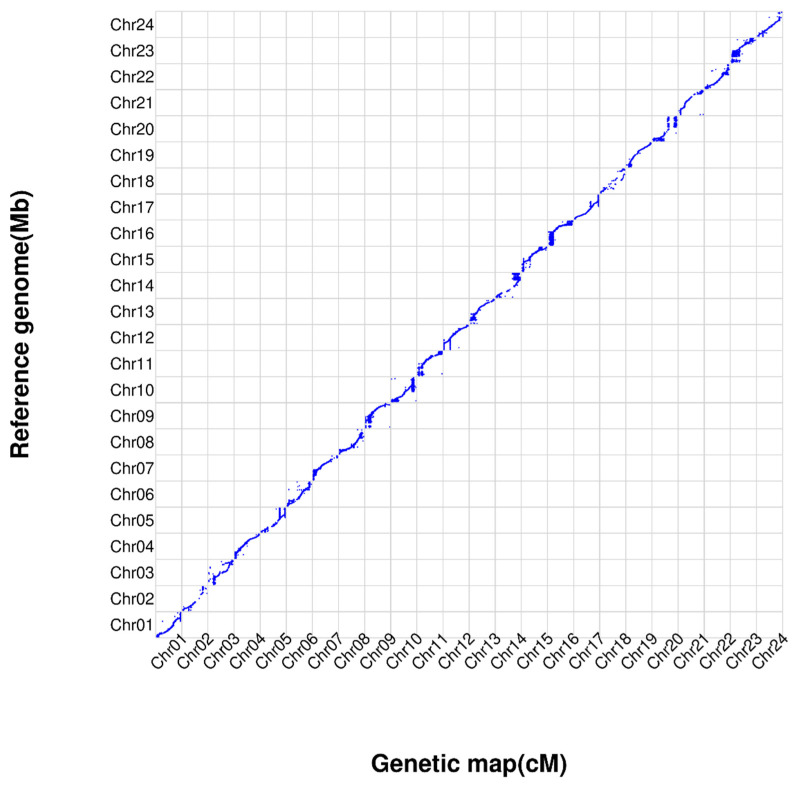
Collinearity analysis between the constructed genetic map and the physical genome of *L. crocea*. The plot compares the order and position of markers on the linkage map (*y*-axis, genetic distance in cM) against their physical positions on the reference genome assembly (*x*-axis, physical distance in Mb).

To evaluate the quality and accuracy of the constructed genetic map, we further analyzed the collinearity between the genetic map and the reference genome of *L. crocea*. By comparing the physical positions of markers on the genome with their corresponding genetic distances on the linkage map, collinearity analysis revealed a strong overall concordance between the two datasets (Figure 4). The marker order within most linkage groups was consistent with the physical order in the genome, with no large-scale inversions or rearrangements observed. Minor local discrepancies in marker order may be attributed to genomic assembly gaps or recombination hotspots. These results confirm the high accuracy and reliability of the high-density genetic map constructed in this study, providing a robust framework for subsequent fine-scale QTL mapping.

### 2.4. QTL Mapping for Growth-Related Traits

**Figure 5:**
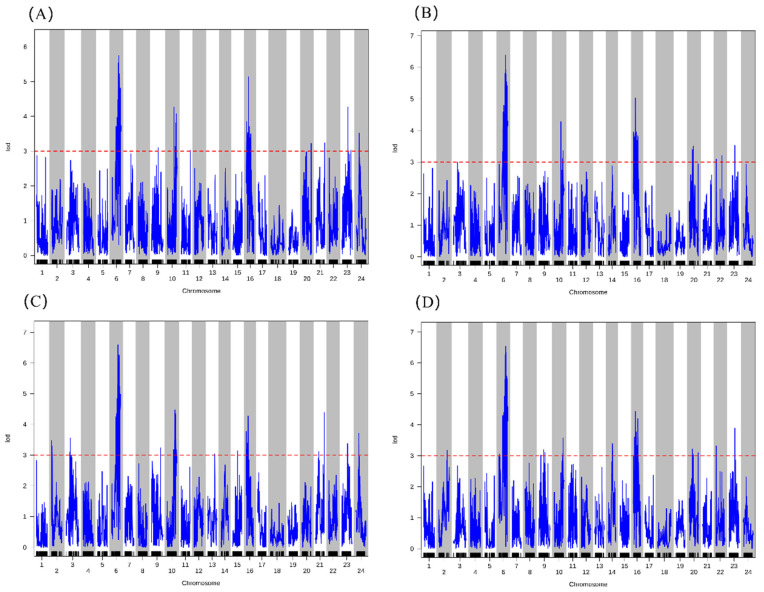
Genome-wide QTL mapping profiles for key growth-related traits in *L. crocea*. (**A**) Total length (AL). (**B**) Body length (BL). (**C**) Body weight (Wt). (**D**) Body height (CH). The *x*-axis represents the linkage group (LG) number, and the *y*-axis shows the LOD (logarithm of odds) score. The horizontal dashed line (red) indicates the genome-wide significance LOD threshold (≥3.0), determined by 1000 permutation tests. Blue lines represent the fitted LOD scores for each SNP locus.

QTL mapping for growth-related traits identified a total of 53 significant QTLs (LOD ≥ 3.0) distributed across 16 linkage groups (LGs), pertaining to the yield trait (Wt) and three key morphological traits: AL, BL, and CH (Figure 5). Specifically, Wt was associated with 12 QTLs, each explaining 0.14 to 5.83% of the phenotypic variance (PVE) (Figure 5C). Notably, five of the Wt-related QTLs co-localized with QTLs for AL and/or BL, particularly on LG6, LG10, LG16, and LG23, suggesting shared genetic regulation between weight gain and body elongation. For the length-related traits, AL and BL shared 8 co-localized QTLs, located on LG6 (2 QTLs), LG10 (2 QTLs), LG16 (2 QTLs), LG20, and LG23 (Figure 5A,B). Among these, the QTL on LG6 (20.8–22.1 Mb) showed the strongest effects on both AL (LOD = 5.75, PVE = 2.56%) and BL (LOD = 6.38, PVE = 1.49%), representing a major locus for longitudinal growth (Table 2). CH was regulated by 14 QTLs distributed across eight LGs (Figure 5D). Four of these co-localized with QTLs for AL and BL, all located on LG6 and LG16, indicating genetic regions that influence both body height and length. The most prominent co-localized region was on LG6 (20.4–28.4 Mb), which contained high-effect QTLs for all four traits (Wt, AL, BL, CH), with LOD scores ranging from 3.24 to 6.98 (Table 2).

Furthermore, analysis of the remaining morphological traits, head CL, QL, TH, TL, and WL revealed that QTLs for these traits also co-localized within the same intervals on LG6 and LG16. Notably, the region on LG16 (15.84–16.23 Mb) contained overlapping QTLs for all nine growth-related traits examined, representing a genomic hotspot with pleiotropic effects on overall body morphology. Overall, LG6 and LG16 were identified as genomic hotspots harboring multiple co-localized QTLs for growth-related traits (Table 2 and Table S2). In particular, LG6 contained a cluster of QTLs with major effects on weight, length, and height, suggesting the presence of a key genomic region governing overall growth performance and body conformation in *L. crocea*. QTL mapping results for the remaining morphological traits are provided in Supplementary Figure S1.

### 2.5. QTL Mapping for VWND-Resistance Traits

**Figure 6:**
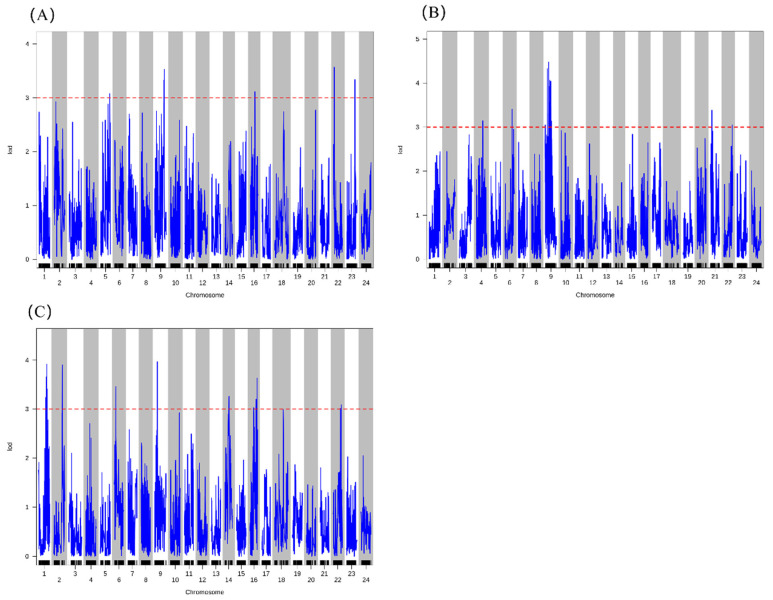
Genome-wide QTL mapping profiles for VWND-resistance traits in *L. crocea*. (**A**) Survival time after bacterial challenge (AT). (**B**) Relative abundance of *P. plecoglossicida* in liver (PPLL). (**C**) Relative abundance of *P. plecoglossicida* in spleen (PPSL). The *x*-axis represents the linkage group (LG) number, and the *y*-axis shows the LOD score. The horizontal dashed line (red) indicates the genome-wide significance LOD threshold (≥3.0). Blue lines represent the fitted LOD scores for each SNP locus.

A total of 20 significant QTLs were identified for the three disease resistance traits: survival time after challenge (AT), relative pathogen load in spleen (PPSL), and relative pathogen load in liver (PPLL) (Figure 6). AT was associated with five QTLs distributed across LG5, LG9, LG16, LG22, and LG23 (Figure 6A), with PVE values ranging from 2.69% to 8.93%. The QTL on LG9 (32.11–32.21 Mb) showed the strongest effect (PVE = 8.93%, LOD = 3.52) (Table 3). For pathogen load traits, eight QTLs were detected for PPSL on LG1, LG2, LG6, LG9, LG14, LG16, and LG22, explaining 1.80–5.58% of phenotypic variance (Figure 6B). The QTLs on LG1 (20.48–20.67 Mb) and LG2 (21.62–22.79 Mb) exhibited the highest PVE values (5.58% and 5.52%, respectively). Seven QTLs were associated with PPLL, located on LG4, LG6, LG9, LG21, and LG22, with PVE ranging from 0.78% to 4.87% (Figure 6C). The most significant PPLL QTL was mapped to LG9 (27.18–27.28 Mb; LOD = 4.04, PVE = 4.19%). Notably, several genomic regions exhibited the co-localization of QTLs for multiple resistance traits. LG9 contained QTLs for all three traits within three distinct intervals: 16.85–17.02 Mb (affecting PPSL), 21.30–27.27 Mb (affecting PPLL), and 32.11–32.21 Mb (affecting AT). On LG16, QTLs for AT and PPSL were clustered within three adjacent intervals (20.17–20.25 Mb, 21.26–21.41 Mb, and 23.88–25.21 Mb). On LG22, overlapping QTLs for AT, PPLL, and PPSL were identified within the region 0.34–2.62 Mb (Table 3 and Table S2). In contrast, no overlapping QTLs were observed between PPLL and AT in other genomic regions, indicating that liver pathogen load may be regulated independently of survival duration under challenge conditions.

### 2.6. Co-Localization of Growth and Disease Resistance

To investigate the genetic relationship between growth performance and VWND resistance, we analyzed the co-localization of QTLs identified for both trait categories. Two genomic regions were found to harbor co-localizing QTLs influencing growth and disease resistance traits (Table 4). On LG16, a QTL interval spanning 21.36–21.41 Mb was shared between AL and AT, explaining 3.37% and 3.57% of PVE, respectively. Within this interval, a single annotated gene, *Unc5d*, was identified. *Unc5d* encodes a netrin receptor involved in axon guidance and has been implicated in immune modulation and cellular stress responses in vertebrates. On LG22, a QTL region between 1.48 and 1.68 Mb co-localized for BL and AT. This locus accounted for 0.66% of phenotypic variance in BL but exerted a stronger effect on AT, explaining 5.99% of variance. Two genes, *SCN5A* and *HUS1*, were annotated within this region. *SCN5A* encodes a voltage-gated sodium channel subunit associated with cardiac electrophysiology and stress adaptation, while *HUS1* is a component of the DNA damage checkpoint complex involved in cellular senescence and genomic integrity maintenance.

## 3. Discussion

This study constructed a high-density SNP-based genetic map for *L. crocea* and performed systematic QTL mapping for two economically vital growth trait categories: yield (body weight, Wt) and morphological traits (e.g., AL, BL, CH) as well as resistance traits against visceral white nodules disease (VWND). The high-resolution map enabled precise dissection of the genetic architecture underlying these traits. Overall, our results revealed strong genetic integration within growth traits, partial independence among resistance components, and largely separate genetic control between growth and disease resistance, with limited QTL co-localization. These findings provide important insights for breeding programs aiming to simultaneously improve yield, morphology, and disease resistance in *L. crocea*.

### 3.1. Phenotypic Correlations Reflect Integrated Growth Traits and Distinct Components of Disease Resistance

The traits analyzed in this study—nine growth-related and three disease resistance-related traits—all showed continuous variation, fitting their quantitative genetic nature. Strong positive correlations were observed among all morphological traits and between these traits and the yield trait (Wt), aligning with findings in other fish species [6,24]. This integration likely reflects the coordinated development program governing body size prior to sexual maturity. Among the disease resistance traits, survival time (AT) was positively correlated with splenic pathogen load (PPSL), consistent with our earlier report [21]. AT is a widely adopted and stable phenotypic indicator that integrates the host’s overall physiological and immune response [25,26]. In contrast, the weak correlation between liver (PPLL) and spleen (PPSL) pathogen loads suggests organ-specific infection dynamics, possibly due to differences in local immunity, metabolism, or tissue tropism of *P. plecoglossicida* [27].

Notably, the yield trait (Wt) and all morphological traits showed moderate positive correlations with AT and weak links to PPSL, but no significant association with PPLL. This is consistent with previous findings in Mekong striped catfish (*Pangasianodon hypophthalmus*), where growth performance was reported to have a moderate favorable genetic correlation with survival under pathogen challenge while showing weak correlations with pathogen susceptibility-related traits [28]. This indicates that faster-growing individuals may survive longer under challenge and tolerate slightly higher splenic pathogen loads, without a corresponding rise in hepatic infection. Similar organ-specific pathogen load patterns have been observed in various aquatic and terrestrial hosts, where splenic pathogen accumulation can be dissociated from hepatic infection during the progression of disease, reflecting differential organ-specific immune responses to pathogens [29,30]. In farming environments, individuals with higher growth rates may possess better overall vigor or stress tolerance, contributing to extended survival despite infection. This phenomenon has been widely documented in aquaculture species, including *Labeo rohita* [31] and *Oreochromis niloticus* [32], where enhanced growth performance is often associated with improved disease resistance and stress resilience, either through intrinsic physiological vigor or better resource allocation for immune defense [33].

### 3.2. A High-Density SNP Genetic Map Enables Accurate QTL Mapping in L. crocea

The genetic map constructed in the present study had an average marker interval of 0.051 cM, representing one of the densest linkage maps developed for a marine fish [20,22,23]. High heterozygosity and environmental sensitivity often constrain genetic studies in aquaculture species, as these factors can lead to unstable phenotypic measurements and reduced accuracy of genetic linkage analysis [34,35]. The success of this map demonstrates the utility of whole-genome resequencing combined with a bin-marker strategy, an approach applicable to other aquatic organisms with complex genomes. This strategy has been successfully employed in other marine and freshwater fish species, such as *Mylopharyngodon piceus* [36] and *L*. *polyactis* [23], to overcome genome complexity and construct high-quality genetic maps.

High map density directly improved QTL detection, as dense marker coverage minimizes the genetic distance between markers and target QTLs, thereby reducing the probability of marker-QTL recombination and enhancing the efficiency of QTL localization [37]. All 12 traits yielded significant QTLs, with PVE ranging from 0.14% to 8.93%. The wide range of PVE values suggests that these traits are controlled by multiple minor-effect QTLs, which is consistent with the quantitative genetic characteristics of most economic traits in aquaculture species [38]. Similar findings have been reported in previous QTL studies on aquatic economic traits. Ali et al. [39] detected growth-related QTLs with relatively low PVEs in rainbow trout, and Liu et al. [40] reported analogous results (PVEs ranging from 2.2% to 4.1%) for disease resistance traits in Asian seabass, both indicating that these quantitative traits are regulated by multiple minor effect QTLs, which is consistent with our study results. The efficient detection of these minor effect QTLs in our study, even with PVEs as low as 0.14%, further highlights the advantage of our high-density genetic map combined with precise phenotyping in dissecting the genetic basis of complex quantitative traits in aquaculture species [36].

### 3.3. QTL Co-Localization Indicates Largely Independent Genetic Control of Growth and Disease Resistance

A key finding of this study is the general genetic independence between growth traits (both yield and morphology) and VWND resistance. Among the 53 growth-related and 20 resistance-related QTLs we identified, only two genomic intervals exhibited co-localization between morphological traits (AL or BL) and AT. Crucially, the QTLs with the largest effects on growth (e.g., on LG6 and LG16) and those with the largest effects on resistance (e.g., on LG9 and LG22) mapped to distinct linkage groups, indicating that the major genetic determinants for these trait complexes are separate.

Furthermore, this pattern of limited genetic overlap is not unique to *L. crocea*. In agreement with our findings, studies in other aquaculture species have reported similar dissociations. For instance, in *Litopenaeus vannamei*, genetic correlations between growth traits and resistance to white spot syndrome virus are typically low, enabling independent selection for these traits [41,42]. Similarly, in *Salmo salar*, genome-wide association studies have identified largely distinct genomic regions controlling growth and resistance to sea lice or pancreas disease, with minimal locus overlap [43]. Moreover, we observed that the genetic pattern aligned well with the phenotypic correlation results. In this study, the moderate correlation between growth and AT may be attributed to a limited number of shared quantitative trait loci (QTLs). Conversely, the lack of correlation with PPLL/PPSL suggests that there are no overlapping QTLs between the two traits. Furthermore, even when the QTLs associated with growth and resistance are located on the same linkage group, they are often situated at considerable physical distances from one another, indicating a high probability of recombination. This further supports the notion of relatively independent regulatory mechanisms governing these traits. Similar genetic mechanisms have been extensively documented in plants [44,45]. In wheat and other cereal crops, overlapping QTLs governing growth and disease resistance traits are exceedingly rare [46]. Even when they are found within the same linkage group, the physical distance between the QTL intervals is typically substantial.

### 3.4. Candidate Genes in Co-Localized Regions Support a Model of Weak Pleiotropy and Predominantly Independent Genetic Control

From a breeding perspective, this largely independent genetic control indicates that simultaneous improvement of yield, morphology, and VWND resistance is feasible. However, it is still necessary to closely monitor a few co-localized regions (such as LG16 and LG22) to manage potential trait interactions during the selection process. In the present study, the annotated candidate genes within these regions, *Unc5d* on LG16 and *SCN5A* and *HUS1* on LG22, are not canonical regulators of core growth (e.g., components of the somatotropic axis) or canonical immune pathways (e.g., pattern recognition receptors or inflammatory cytokines) [47,48]. Instead, their known functions are associated with fundamental cellular and systemic processes: neuronal guidance and cellular stress response (*Unc5d*) [49], electrochemical signaling and systemic stress adaptation (*SCN5A*) [50], and genomic integrity maintenance (*HUS1*) [51].

The phenotypic variance explained (PVE) by these co-localizing QTLs further indicates their modest and distinct contributions. The locus on LG16 exhibited comparable effects on total length (AL, PVE = 3.37%) and survival time (AT, PVE = 3.57%). In contrast, the locus on LG22 had a minimal impact on body length (BL, PVE = 0.66%) but a more pronounced effect on survival time (AT, PVE = 5.99%). This pattern is consistent with a model of weak or trait-specific pleiotropy, where genetic variants exert minor, potentially indirect, effects on multiple traits, or where linked genes with distinct primary functions are localized within the same genomic interval [52]. Moreover, the absence of major growth or immune effector genes within co-localization intervals suggests that the observed phenotypic correlations are unlikely to be driven by strong, direct pleiotropy of key developmental or immunological pathways. This finding aligns with reports in other aquaculture species where growth and disease resistance QTLs largely map to distinct genomic regions [41,42,43]. Instead, the shared genetic influence may stem from variants in genes governing systemic homeostasis, basal stress responses, or fundamental cellular functions, which secondarily modulate both growth and defense phenotypes [53]. Collectively, these interpretations reinforce the genome-wide pattern observed here: the vast majority of QTLs for yield, morphology, and disease resistance are genetically distinct. Therefore, the functional annotation and modest effect sizes of the candidate genes within the co-localized QTLs corroborate the conclusion that growth and VWND resistance in *L. crocea* are primarily under independent genetic control. The identified loci likely represent peripheral, integrative nodes rather than central genetic constraints, a genetic architecture that is favorable for breeding programs aiming to achieve simultaneous genetic gain in both trait complexes without encountering severe antagonistic selection responses [54].

### 3.5. Study Limitations and Future Perspectives

This study represents an important step toward understanding the genetic relationship between growth and disease resistance in *L. crocea* through high-density linkage mapping and QTL analysis. However, certain considerations should be taken into account when extrapolating these findings. First, as a single-family study, the QTLs detected herein represent those segregating in this specific family and may not capture all genetic variants influencing growth and disease resistance in the broader population. This is consistent with findings in other species where QTL effects can vary across genetic backgrounds [16]. Nevertheless, single-family QTL mapping remains a valuable approach for initial genetic dissection, and the consistency of our findings with phenotypic correlations supports their biological relevance. Second, most QTLs identified in this study explained relatively low proportions of phenotypic variance (PVE < 5%), consistent with the polygenic architecture typical of complex traits in aquaculture species. Nevertheless, the modest sample size may limit statistical power to detect QTLs with very small effects, and some genuine loci may have escaped detection. Third, although permutation tests were employed to control genome-wide false positive rates-a widely accepted approach in QTL mapping studies [55]. We acknowledge that additional false discovery rate (FDR) control was not applied. Permutation tests were chosen because they account for the correlation structure among linked markers, which FDR procedures may not fully capture [56], and this approach is widely adopted in high-density QTL mapping studies in aquaculture species [17,22,23]. Nevertheless, FDR control could provide complementary information, particularly for prioritizing candidate loci for future validation. Fourth, while candidate genes were identified within co-localized intervals based on genomic annotation, their functional roles in growth and disease resistance remain to be experimentally validated. Future studies incorporating transcriptomic, proteomic, or gene editing approaches will be essential to confirm their biological relevance and facilitate the development of marker-assisted selection programs for simultaneous improvement of growth and disease resistance in *L. crocea* breeding.

## 4. Materials and Methods

### 4.1. Experimental Materials and Full-Sib Family Construction

The experimental fish used in this study were derived from the Daiqu strain of *L. crocea*. Five full-sibling families were first established via artificial insemination at Xiangshan Harbor Aquatic Hatchery Co., Ltd. (Ningbo, China) in April 2023, following the method described by Ye et al. [21]. Briefly, 10 sexually mature females and 5 males (18 months old, hybrids of wild and farmed populations) were injected with luteinizing hormone-releasing hormone A3 (LHRH-A3) (Ningbo second hormone factory, Ningbo, China) at doses of 1 μg/fish and 0.5 μg/fish (dissolved in 1 mL sterile saline), respectively, for spawning induction. After artificial insemination at a gamete ratio of 1:1, five full-sibling lineages were successfully obtained. A family was selected for the present study. A total of 150 healthy individuals from this family at 8 months of age (average body weight: 22.1 ± 4.2 g) were selected for the bacterial challenge experiment.

### 4.2. Phenotypic Trait Determination and Data Quality Control

#### 4.2.1. Determination of Yield Trait and Morphological Traits

Nine growth-related traits were determined for each individual, including 1 yield trait and 8 morphological traits: yield trait was body weight (Wt, directly reflecting aquaculture yield); morphological traits included total length (AL), body length (BL), head length (CL), body height (CH), tail height (TH), trunk length (TL), caudal peduncle length (QL), and caudal fin length (WL). These parameters were measured using an automated phenotyping acquisition system as described by Wang et al. [57]. Each trait was independently measured three times by the same operator, and the average value was taken as the final phenotypic value to reduce measurement errors.

#### 4.2.2. Determination of VWND Resistance-Related Traits

The bacterium (*P. plecoglossicida*) used in this study was isolated from *L. crocea* affected by a natural outbreak in Xiangshan. Based on 16S rRNA gene (16S) homology and biochemical tests, it was classified as the XSDHY-P strain [58]. The bacterial challenge experiment was performed following the method described by Ye et al. [21] with identical operational procedures. Briefly, bacteria were cultured overnight in TSA liquid medium at 28 °C with 200 rpm shaking for 24 h. The infection concentration was set to 1 × 10^5^ CFU/mL, based on the 96 h LC_50_ previously verified by our team (unpublished data), to ensure high infection success rate and uniformity. A total of 150 experimental fish were intraperitoneally injected with 0.5 mL of the bacterial suspension at the fin base, and the entire injection process was completed within 30 min with water temperature stably maintained. A control group (*n* = 30) receiving 0.5 mL sterile phosphate-buffered saline (PBS) was included in parallel, and no mortality was observed in the control group throughout the experiment.

Three VWND resistance-related traits were determined: survival time (AT), the relative abundance of *P. plecoglossicida* in spleen (PPSL), and the relative abundance of *P. plecoglossicida* in liver (PPLL). Immediately post-injection, all experimental fish were placed under continuous observation. Fish exhibiting a moribund state (defined as complete unresponsiveness to external stimuli) were promptly removed, and the time of removal was recorded as individual survival time (AT). Subsequently, spleen and liver tissues were aseptically dissected on ice, immediately flash-frozen in liquid nitrogen for subsequent pathogen load quantification (PPLL and PPSL), and a fin clip was collected and preserved in absolute ethanol. Observation continued until survival time data had been recorded for all 150 individuals, at which point the experiment was terminated. The identification methods for PPSL and PPLL followed our previously established protocol [21]. Briefly, total DNA was extracted from the entire spleen and liver tissues using the standard phenol-chloroform method. After quality verification by 1% agarose gel electrophoresis, DNA concentration was normalized to 50 ng/μL using a Nanodrop One spectrophotometer (Thermo Fisher Scientific, China). Quantitative PCR (qPCR) was performed with species-specific primers for *P. plecoglossicida* (pps-F: GGTACTCACCTGGTTGGGCTT; pps-R: GCTTGTCCTTGGTCTGGGAG) and the large yellow croaker single-copy gene gdf8 (gdf8-F: TGGGAGATGACAACAGG; gdf8-R: GCACCGACCAATACACT) as an internal reference. The thermal cycling conditions comprised an initial denaturation at 95 °C for 1 min, followed by 40 cycles of 95 °C for 5 s, 60 °C for 10 s, and 72 °C for 15 s. Relative pathogen load was calculated using the 2^−^ΔΔCt method to quantify *P. plecoglossicida* proliferation per unit of host tissue. Environmental conditions during the experiment were strictly controlled (water temperature 17~18 °C, salinity fluctuation ± 1‰, dissolved oxygen ≥ 6 mg/L) to ensure consistent challenge pressure.

### 4.3. SNP Genotyping and Bin Marker Filter

Genomic DNA was extracted from tissue (fin, spleen, liver) using a commercial DNA extraction kit (Tiangen Biotech, Beijing, China) according to the manufacturer’s instructions. DNA integrity was verified by 1% agarose gel electrophoresis, purity was detected by Nanodrop 2000 (NanoDrop, Waltham, MA, USA), and concentration was measured by Qubit 2.0 fluorometer (Invitrogen, Carlsbad, CA, USA). DNA samples meeting the quality requirements were used for subsequent SNP genotyping.

SNP genotyping was performed on the Illumina NovaSeq 6000 platform (Illumina, San Diego, CA, USA) using whole-genome resequencing technology. The library construction process was as follows: genomic DNA was fragmented by ultrasonic disruption, the fragmented products were end-repaired and ligated to barcoded adapters, the ligated products were amplified by PCR, and fragments of 300~350 bp were selected to construct the library. After quality inspection, the library was subjected to paired-end sequencing (PE150). Raw sequencing data were filtered to remove adapter-contaminated sequences, low-quality sequences (Q < 20), and sequences with N content > 5%. The filtered clean reads were aligned to the reference genome of the *L. crocea* Daiqu strain (GenBank accession number: PRJNA1168280) using BWA software (version 0.7.17), and SNP detection was performed using GATK software (version 4.3.0.0) [59]. High-quality SNPs were filtered under the following criteria: missing rate < 20%, minor allele frequency (MAF) > 0.05, and Hardy–Weinberg equilibrium *p* > 0.001. Prior to genetic map construction, SNP markers were subjected to bin mapping instead of direct use. Briefly, SNPs were first imputed and corrected: a 15-SNP sliding window with a 1-SNP step size was used for chromosome scanning; windows with no less than 13 SNPs typed as “aa” or “bb” were classified as aa or bb, respectively, and other types were imputed and corrected to “ab”. Bins were then defined as continuous non-recombinant SNP clusters with identical genotypes in offspring. Bins shorter than 10 kb and those with severe segregation distortion (chi-square test, *p* < 0.0001) were filtered out. The genetic material origin of bin markers in offspring was visualized by graphical genotype analysis, and the resulting bins were used as polymorphic markers for genetic map construction.

### 4.4. High-Density Genetic Map Construction and QTL Mapping

A high-density genetic map was constructed using Lepmap3 software (version 0.2) based on the double pseudo-testcross strategy and the filtered bin markers. Maternal-specific markers (lm × ll) and paternal-specific markers (nn × np) were grouped with LOD thresholds of 3.0 and a maximum recombination rate of 0.4. Marker order was optimized using the Regression Mapping method, and genetic distances between markers were calculated by the Kosambi mapping method [60]. Shared markers (hk × hk) were used as anchors to integrate parent-specific linkage maps into a consensus map. The quality of the linkage map was assessed using metrics including the number of linkage groups, maximum marker spacing, recombination relationship evaluation, and genomic collinearity.

Based on the high-density genetic map and phenotypic data of the F1 generation, QTL mapping was performed for 9 growth-related traits and 3 disease resistance-related traits using the Interval Mapping (IM) method in MapQTL 6.0 software (Kyazma B.V., Wageningen, The Netherlands). The parameter settings were as follows: step size of 0.1 cM and scanning range covering all linkage groups. The LOD threshold for significant QTLs was determined by 1000 permutation tests (significance level α = 0.05), a widely accepted approach for controlling genome-wide type I error in QTL mapping studies [55]. Permutation tests accounted for the multiple testing inherent in genome-wide scans by generating empirical significance thresholds based on the actual data structure without relying on assumptions about the number of independent tests. QTLs with LOD values ≥ 3 were considered significant.

### 4.5. Data Statistics and Visualization

All data processing and statistical analyses were performed using R software (version 3.5.0) and SPSS 26.0 software (IBM Corp., Armonk, NY, USA) on phenotypic data from 150 experimental samples. Pearson or Spearman correlation coefficients were used for trait correlation analysis, with significance verified by the *t*-test. Result visualization was implemented using R packages (version 3.5.0) and GraphPad Prism 9.0 (GraphPad Software, Inc., Boston, MA, USA): boxplots were used to display QTL mapping results, phenotypic differences, and marker effects; scatter plots and heatmaps were used to visualize trait correlations.

## 5. Conclusions

This study successfully constructed a high-density SNP genetic map for the large yellow croaker and employed it to systematically dissect the genetic architectures underlying yield, morphology, and resistance to VWND. The ultra-fine map resolution enabled precise QTL detection, revealing a landscape where the genetic control of growth and disease resistance is largely distinct. The strong phenotypic and genetic integration within yield and morphological traits contrasts with the limited number of co-localized QTLs shared between growth and disease resistance. The candidate genes found within these rare co-localization intervals further support a model of weak pleiotropy, primarily involving systemic stress response pathways rather than direct regulatory hubs for growth or immunity. Our primary conclusion is that the genetic correlation between growth (both yield and morphology) and VWND resistance in *L. crocea* appears to be weak within this F1 family, consistent with largely independent polygenic architectures. This finding is of significant practical importance as it suggests that antagonistic selection responses are not an inevitable constraint. Breeders can therefore prioritize selection for high yield, desirable morphology, and enhanced disease resistance concurrently, accelerating the development of balanced, high-performance strains. The identified QTL hotspots and candidate genes provide valuable targets for marker-assisted selection and future functional studies.

## Figures and Tables

**Table 1:** Basic information statistics of genetic maps.

Chrom ID	Linkage Group	Marker Count	Total cM	Average cM
Chr01	LG1	1810	66.337	0.037
Chr02	LG2	809	64.825	0.080
Chr03	LG3	1497	76.749	0.051
Chr04	LG4	1862	62.002	0.033
Chr05	LG5	1525	59.417	0.039
Chr06	LG6	1466	56.350	0.038
Chr07	LG7	1580	54.030	0.034
Chr08	LG8	1602	56.335	0.035
Chr09	LG9	1578	68.349	0.043
Chr10	LG10	1564	60.004	0.038
Chr11	LG11	1540	54.355	0.035
Chr12	LG12	1636	56.671	0.035
Chr13	LG13	1442	56.010	0.039
Chr14	LG14	1047	45.715	0.044
Chr15	LG15	1663	55.335	0.033
Chr16	LG16	1344	46.002	0.034
Chr17	LG17	1280	51.001	0.040
Chr18	LG18	377	82.430	0.219
Chr19	LG19	1319	58.678	0.045
Chr20	LG20	910	56.191	0.062
Chr21	LG21	1289	54.033	0.042
Chr22	LG22	1331	55.002	0.041
Chr23	LG23	1110	61.674	0.056
Chr24	LG24	848	59.675	0.070
Total	24	32,429	1417.17	0.051

**Table 2:** Statistics of growth-related QTL mapping information.

Traits	LG	Physical Positions (bp)	Genetic Intervals (cM)	LOD	PVE
AL	LG6	20808759–22129259	0.333	5.75	2.56
		24693467–28394587	4.005	3.74	1.30
	LG9	28799336–28991563	0	3.10	1.25
	LG10	15500932–15602041	0	4.27	0.92
		23964252–24305153	0	4.08	0.61
	LG11	2281308–29418422	0.333	3.02	1.56
	LG16	9923981–14248128	2.334	3.84	1.94
		15844715–16226575	0.333	5.13	1.64
		21360589–21408574	1	3.50	3.37
	LG20	16485080–16783154	0	3.22	4.36
	LG21	28804866–29078714	0.667	3.24	2.60
	LG23	16886521–17746638	0.334	4.26	2.41
		20696553–22135450	0.334	3.02	1.55
	LG24	3171840–3872176	1.667	3.52	1.47
BL	LG6	20808759–22129259	0.333	6.38	1.49
		24693467–28394587	4.005	4.29	1.38
	LG10	15500932–15602041	0	4.28	5.21
		23964252–24305153	0	3.36	1.99
	LG16	9923981–14248128	2.334	3.95	1.80
		15844715–16226575	0.333	5.03	1.51
		20964901–21197665	1.333	3.83	3.29
	LG20	4906981–5110891	0.667	3.50	1.38
	LG22	1475866–1684366	0.333	3.09	0.66
		12918125–12998945	0	3.21	4.87
	LG23	16886521–17746638	0.334	3.52	2.73
CH	LG1	932523–1106946	0	3.01	3.08
	LG3	10958159–10988837	0	3.58	2.65
	LG5	2975985–4145543	0	3.22	2.07
	LG6	20429686–22129259	1.333	6.98	0.45
		24693467–28394587	4.005	4.58	1.08
	LG10	15500932–15602041	0	3.81	3.71
		23964252–24305153	0	3.33	1.15
	LG16	9923981–14248128	2.334	3.82	1.87
		15844715–16226575	0.333	5.04	0.99
		20964901–21197665	1.333	3.20	2.13
	LG20	783856–4793242	0.667	3.88	1.48
		16485080–16783154	0	3.04	2.56
	LG22	12918125–12998945	0	3.32	3.30
	LG23	16886521–17746638	0.334	3.44	1.29
Wt	LG2	1393408–1425256	2	3.46	5.83
	LG3	10958159–10988837	0	3.55	5.43
	LG6	10988837–20891859	0.667	6.59	0.81
		23951204–24082864	0.667	5.24	0.53
	LG9	30441812–31861358	0.667	3.24	4.79
	LG10	19675372–19789011	1	4.47	1.11
	LG13	26329468–26497586	1.667	3.03	2.82
	LG15	25223290–25278782	0	3.14	2.38
	LG16	9923981–14248128	2.334	3.77	2.15
		15844715–16226575	0.333	4.27	0.86
	LG21	18180302–18291324	0.666	3.11	1.43
		28804866–29078714	0.667	4.38	0.14
	LG23	16886521–17746638	0.334	3.37	1.76
	LG24	3171840–3872176	1.667	3.70	2.70
co-localization	LG6	10988837–22129259			0.45–2.56
	LG16	9923981–14248128			1.80–2.15
		15844715–16226575			0.86–1.64
	LG23	16886521–17746638			1.29–2.73

“Physical positions” are based on the reference genome assembly; “Genetic intervals” represent linkage map distances calculated using the Kosambi mapping function.

**Table 3:** Statistics of VWND-resistant related QTL mapping information.

Traits	LG	Physical Positions (bp)	Genetic Intervals (cM)	LOD	PVE
AT	LG5	21745338–22452793	3.334	3.07	5.44
	LG9	32107638–32214739	1	3.52	8.93
	LG16	21262862–21408574	1	3.11	3.57
	LG22	336231–2605973	0.667	3.57	5.99
	LG23	20336210–20385009	0	3.34	2.69
PPLL	LG4	29379938–29506982	0.666	3.14	3.53
	LG6	22259958–22293037	0.333	3.41	3.58
	LG9	2130154–2554995	0.667	3.05	0.89
		22339892–22865611	0.333	4.48	0.78
		27182961–27276350	0	4.04	4.19
	LG21	8265012–8668464	0.666	3.34	3.50
	LG22	17296736–26220045	0.333	3.05	4.87
PPSL	LG1	20482703–20666842	0.334	3.92	5.58
	LG2	21623051–22790636	1.667	3.90	5.52
	LG6	3573844–7021320	1	3.46	4.18
	LG9	16851739–17026950	0	3.97	1.80
	LG14	1324569–20670620	13.684	3.26	2.46
	LG16	20178322–20245992	2.001	3.03	2.21
		23880199–25211027	1.667	3.63	2.55
	LG22	17108204–19788639	0.333	3.09	4.19
Co-localization	LG22	336231–2605973			4.19–5.99

“Physical positions” are based on the reference genome assembly; “Genetic intervals” represent linkage map distances calculated using the Kosambi mapping function.

**Table 4:** Statistics on co-localization information of growth and disease resistance.

Traits	LG	Position	PVE	Target Gene	Pathway
AL-AT	LG16	21360589–21408574	3.37/3.57	*Unc5d*	Axon guidance
BL-AT	LG22	1475866–1684366	0.66/5.99	*SCN5A*	Adrenergic signaling in cardiomyocytes
				*HUS1*	Cellular senescence

## Data Availability

The data presented in this study have been submitted to NCBI with the following accession number: PRJNA1168280. All other data are contained within the main manuscript.

## Supplementary Materials

The following supporting information can be downloaded at: https://www.mdpi.com/article/10.3390/ijms27062531/s1.

## References

[1] Li Z.Y., Fang M., Tang X., Zhang D.L., Wang Z.Y. (2023). Disentangling genetic variation for endurance and resistance to visceral white-nodules disease in large yellow croaker (*Larimichthys crocea*) using genome information. Aquaculture.

[2] Li C., Wang S., Ren Q., He T., Chen X. (2020). An outbreak of visceral white nodules disease caused by *Pseudomonas plecoglossicida* at a water temperature of 12 °C in cultured large yellow croaker (*Larimichthys crocea*) in China. J. Fish Dis..

[3] Antonello J., Massault C., Franch R., Haley C., Pellizzari C., Bovo G., Patarnello T., Koning D.J., Bargelloni L. (2009). Estimates of heritability and genetic correlation for body length and resistance to fish pasteurellosis in the gilthead sea bream (*Sparus aurata* L.). Aquaculture.

[4] Moode V.K., Puchakayala M., Gannavarapu S.K., Kommana M., Krishna L., Lekkala S., Chakravartty N., Lachagari V., Umar S.N., Akkareddy S. (2025). Genetic analysis of trade-offs among disease resistance, yield, and quality traits employing genome-wide association mapping in indica rice (*Oryza sativa* L.). Mol. Breed..

[5] Deng Y.W., Zhai K.R., Xie Z., Yang D.D., Zhu X.D., Liu J.Z., Wang X., Qin P., Yang Y.Z., Zhang G.M. (2017). Epigenetic regulation of antagonistic receptors confers rice blast resistance with yield balance. Science.

[6] Gjedrem T., Baranski M. (2010). Selective Breeding in Aquaculture: An Introduction.

[7] Song H.L., Xu S.J., Luo K., Hu M., Luan S., Shao H., Kong J., Hu H.X. (2022). Estimation of genetic parameters for growth and egg related traits in Russian sturgeon (*Acipenser gueldenstaedtii*). Aquaculture.

[8] Gonçalves T.G., Ataides K.S., Carvalheiro R., Nova F.A.P.C., Neto R.V.R. (2024). Genetic parameter estimates indicate the possibility of genetic gain by selecting for reproductive traits of females from a commercial tilapia population (*Oreochromis niloticus*). Aquaculture.

[9] Yu X.X., Adnoy T., Lv Z.M., Wu C.W., Gjoen H.M. (2020). Phenotypic and genetic parameter estimation for growth traits in juvenile large yellow croaker (*Larimichthys crocea*). Fish. Aquac. J..

[10] Yang Z.Y., Lu N.N., Zhai L. (2025). Study on production strategies for marine aquaculture in China at different scales: A case study of large yellow croaker (*Larimichthys crocea*). Aquac. Int..

[11] Zhou Z.X., Han K.H., Wu Y.D., Bai H.Q., Ke Q.Z., Pu F., Wang Y.L., Xu P. (2019). Genome-wide association study of growth and body-shape-related traits in large yellow croaker (*Larimichthys crocea*) using ddRAD sequencing. Mar. Biotechnol..

[12] Wu Y.D., Zhou Z.X., Pan Y., Zhao J., Bai H.Q., Chen B.H., Zhang X.Y., Pu F., Chen J., Xu P. (2021). GWAS identified candidate variants and genes associated with acute heat tolerance of large yellow croaker. Aquaculture.

[13] Liu W., Wang Y.H., Feng M.S., Wang J.Y., Zeng J.J., Deng Y.C., Pu F., Li N., Xu P. (2026). Integrating geometric morphometrics and GWAS to reveal the genetic basis of body shape variation in large yellow croaker (*Larimichthys crocea*). Aquaculture.

[14] Wan L., Wang W.J., Liu G.J., Dong L.S., Li W.B., Han Z.F., Ye K., Wang Z.Y. (2019). A genome-wide association study of resistance to *Pseudomonas plecoglossicida* infection in the large yellow croaker (*Larimichthys crocea*). Aquac. Int..

[15] Jiao W.Q., Fu X.F., Dou J.Z., Li H.D., Su H.L., Mao J.X., Yu Q., Zhang L.L., Hu X.L., Huang X.T. (2014). High-resolution linkage and quantitative trait locus mapping aided by genome survey sequencing: Building up an integrative genomic framework for a bivalve mollusc. DNA Res..

[16] Besnier F., Solberg M.F., Harvey A.C., Carvalho G.R., Bekkevold D., Taylor M.I., Creer S., Nielsen E.E., Skaala Ø., Ayllon F. (2020). Epistatic regulation of growth in Atlantic salmon revealed: A QTL study performed on the domesticated-wild interface. BMC Genet..

[17] Wang X.P., Gao D.D., Zhang G.W., Ge Y.C., Wang X.H. (2025). High-density SNP-based linkage map construction and QTL analysis for growth-related traits in *Luciobarbus brachycephalus* using whole-genome resequencing data. Front. Genet..

[18] Ning Y., Liu X.D., Wang Z.Y., Guo W., Li Y.Y., Xie F.J. (2007). A genetic map of large yellow croaker *Pseudosciaena crocea*. Aquaculture.

[19] Ye H., Liu Y., Liu X.D., Wang X.Q., Wang Z.Y. (2014). Genetic mapping and QTL analysis of growth traits in the large yellow croaker *Larimichthys crocea*. Mar. Biotechnol..

[20] Ao J.Q., Li J., You X.X., Mu Y.N., Ding Y., Mao K.Q., Bian C., Mu P.F., Shi Q., Chen X.H. (2015). Construction of the high-density genetic linkage map and chromosome map of large yellow croaker (*Larimichthys crocea*). Int. J. Mol. Sci..

[21] Ye T., Liu F., Liang X., Guo D.D., Zhan W., Shao G.E., Lou B. (2025). BSA-seq and transcriptome analysis identification candidate genes associated with *Pseudomonas plecoglossicida* resistance in the large yellow croaker (*Larimichthys crocea*). Fish Shellfish Immunol..

[22] Kong S.N., Ke Q.Z., Chen L., Zhou Z.X., Pu F., Zhao J., Bai H.Q., Peng W.Z., Xu P. (2019). Constructing a high-density genetic linkage map for large yellow croaker (*Larimichthys crocea*) and mapping resistance trait against ciliate parasite *Cryptocaryon irritans*. Mar. Biotechnol..

[23] Li Q., Zhu J.J., Liu S.F., Liu H.W., Zhang T.L., Ye T., Lou B., Liu F. (2024). QTL mapping-based identification of visceral white-nodules disease resistance genes in *Larimichthys polyactis*. Int. J. Mol. Sci..

[24] Chen H.L., Si Z.X., Du J.X., Xu X.D., Wang J., Wang C.H. (2019). Correlation and path coefficient analysis of the morphometric traits and body weight for the four color patterns of Oujiang color common carp. Prog. Fish. Sci..

[25] Vu N.T., Phuc T.H., Huong T.T.M., Nguyen N.H. (2026). First high-density linkage map and quantitative trait loci for disease resistance in striped catfish *Pangasianodon hypophthalmus*. Int. J. Mol. Sci..

[26] Su Y.Q., Qu S.Y., Liu Y., Teng J.M., Zeng J.Y., Liu S., Li S.S., Chen S.L. (2026). Genome-wide association study of resistance to infectious spleen and kidney necrosis virus in mandarin fish (*Siniperca chuatsi*). Aquaculture.

[27] Jin L.Y., Li S.Y., Yin F., Tao Z., Xie X., Zhou S.M. (2025). Comparison of the host-pathogen interactions between *Pseudomonas plecoglossicida* and *Nocardia seriolea* in a cell line derived from head kidney of yellow large croaker. Fish Shellfish Immunol..

[28] Pham K.D., Ødegård J., Nguyen S.V., Gjøen H.M., Klemetsdal G. (2021). Genetic correlations between challenge tested susceptibility to bacillary necrosis, caused by *Edwardsiella ictaluri*, and growth performance tested survival and harvest body weight in Mekong striped catfish (*Pangasianodon hypophthalmus*). J. Fish Dis..

[29] Coward C., Restif O., Dybowski R., Grant A.J., Maskell D.J., Mastroeni P. (2014). The effects of vaccination and immunity on bacterial infection dynamics in vivo. PLoS Pathog..

[30] Yang J.Y., Hu X.T., Lv C.J., Cheng C.X., Zhang Q., Sun X.M., Du X.Z., Jin M.L. (2025). Infection characteristics, transcriptomics, and metabolomics of african swine fever virus SY-1 strain in orally infected weaned landrace piglets. Transbound. Emerg. Dis..

[31] Sahu S., Das B.K., Pradhan J., Mohapatra B.C., Mishra B.K., Sarangi N. (2007). Effect of Mangifera indica kernel as a feed additive on immunity and resistance to Aeromonas hydrophila in *Labeo rohita* fingerlings. Fish Shellfish Immunol..

[32] Poolsawat L., Li X.Q., Yang H., Yang P.X., Chowdhury M.A.K., Yusuf A., Leng X.J. (2020). The potentials of fructooligosaccharide on growth, feed utilization, immune and antioxidant parameters, microbial community and disease resistance of tilapia (*Oreochromis niloticus* × *O. aureus*). Aquac. Rep..

[33] Xia Y., Wang M., Gao F.Y., Lu M.X., Chen G. (2020). Effects of dietary probiotic supplementation on the growth, gut health and disease resistance of juvenile Nile tilapia (*Oreochromis niloticus*). Anim. Nutr..

[34] Liu T.G., Guo Y.H., Liu S.S., Li J.F., Ye N.H. (2011). Genetic variability of half-smooth tongue sole *Cynoglossus semilaevis* populations using microsatellite markers. Acta Oceanol. Sin..

[35] Robledo D., Ogwang J., Byakora E., Nascimento-Schulze J.C., Benda K.K., Fraslin C., Salisbury S., Solimo M., Mayega J.F., Peter B. (2024). Genetic diversity and population structure of farmed and wild Nile tilapia (*Oreochromis niloticus*) in Uganda: The potential for aquaculture selection and breeding programs. Genomics.

[36] Guo J.M., Wang A.Q., Mao S.Q., Xu X.Y., Li J.L., Shen Y.B. (2022). Construction of high-density genetic linkage map and QTL mapping for growth performance in black carp (*Mylopharyngodon piceus*). Aquaculture.

[37] Zhu C.K., Liu H.Y., Pan Z.J., Chang G.L., Wang H., Wu N., Ding H.Y., Yu X.S. (2019). Construction of a high-density genetic linkage map and QTL mapping for growth traits in *Pseudobagrus ussuriensis*. Aquaculture.

[38] Vu N.T., Nguyen N.H. (2019). Quantitative genetic changes in reproductive performance of giant freshwater prawn after 10 years of selection for increased growth rate. Reprod. Domest. Anim..

[39] Ali A., Al-Tobasei R., Lourenco D., Leeds T., Kenney B., Salem M. (2020). Genome-wide identification of loci associated with growth in rainbow trout. BMC Genom..

[40] Liu P., Wang L., Wan Z.Y., Ye B.Q., Huang S.Q., Wong S.M., Yue G.H. (2016). Mapping QTL for resistance against viral nervous necrosis disease in Asian seabass. Mar. Biotechnol..

[41] Ren S.J., Prentis P., Mather P.B., Li Y.T., Tang B.G., Hurwood D.A. (2020). Genetic parameters for growth and survival traits in a base population of Pacific white shrimp (*Litopenaeus vannamei*) developed from domesticated strains in China. Aqtaculture.

[42] Sui J., Sun K., Kong J., Tan J., Dai P., Cao J.W., Luo K., Luan S., Xing Q., Meng X.H. (2024). Estimation of genetic parameters for growth and WSSV resistance traits in *Litopenaeus vannamei*. Animals.

[43] Tsai H.Y., Bishop S.C., Houston R.D. (2015). The Genetic Regulation of Growth and Sea Lice Resistance in Farmed Atlantic salmon (S. salar).

[44] Siddique M.I., Silverman E., Louws F., Panthee D.R. (2024). Quantitative trait loci mapping for bacterial wilt resistance and plant height in tomatoes. Plants.

[45] Liu G., Jia Y., Correa-Victoria F.J., Prado G.A., Yeater K.M., McClung A., Correll J.C. (2009). Mapping quantitative trait loci responsible for resistance to sheath blight in rice. Phytopathology.

[46] Poland J., Rutkoski J. (2016). Advances and challenges in genomic aelection for disease resistance. Annu. Rev. Phytopathol..

[47] Paradisi A., Maisse C., Bernet A., Coissieux M.M., Maccarrone M., Scoazec J.Y., Mehlen P. (2008). NF-kappaB regulates netrin-1 expression and affects the conditional tumor suppressive activity of the netrin-1 receptors. Gastroenterology.

[48] Pérez-Sánchez J., Simó-Mirabet P., Naya-Català F., Martos-Sitcha J.A., Perera E., Bermejo-Nogales A., Benedito-Palos L., Calduch-Giner J.A. (2018). Somatotropic axis regulation unravels the differential effects of nutritional and environmental factors in growth performance of marine farmed fishes. Front. Endocrinol..

[49] Wang H., Zhang B., Gu M., Li S., Chi Z.F., Hao L.C. (2014). Overexpression of the dependence receptor UNC5H4 inhibits cell migration and invasion, and triggers apoptosis in neuroblastoma cell. Tumour. Biol..

[50] Kinoshita K., Takahashi H., Hata Y., Nishide K., Kato M., Fujita H., Yoshida S., Murai K., Mizumaki K., Nishida K. (2016). SCN5A(K817E), a novel Brugada syndrome-associated mutation that alters the activation gating of NaV1.5 channel. Heart Rhythm..

[51] Cotta-Ramusino C., McDonald E.R., Hurov K., Sowa M.E., Harper J.W., Elledge S.J. (2011). A DNA damage response screen identifies RHINO, a 9-1-1 and TopBP1 interacting protein required for ATR signaling. Science.

[52] Solovieff N., Cotsapas C., Lee P., Purcell S.M., Smoller J.W. (2013). Pleiotropy in complex traits: Challenges and strategies. Nat. Rev. Genet..

[53] Rauw W.M. (2012). Immune response from a resource allocation perspective. Front. Genet..

[54] Hill W.G. (2014). Applications of population genetics to animal breeding, from Wright, Fisher and Lush to genomic prediction. Genetics.

[55] You X., Shan X., Shi Q. (2020). Research advances in the genomics and applications for molecular breeding of aquaculture animals. Aquaculture.

[56] Kaler A.S., Gillman J.D., Beissinger T., Purcell L.C. (2020). Comparing different statistical models and multiple testing corrections for association mapping in soybean and maize. Front. Plant Sci..

[57] Wang R., Zhu J.J., Liu S.F., Ye T., Guo D.D., Liu Y., Lou B., Liu F. (2026). Genetic parameter estimation of growth traits and their implications for selective breeding in small yellow croaker (*Larimichthys polyactis*). Mar. Biotechnol..

[58] Tao Z., Wang G.L., Zhou S.M. (2018). Complete genome sequence of *Pseudomonas plecoglossicida* XSDHY-P, a strain that is pathogenic for the marine fish *Larimichthys crocea*. Microbiol. Resour. Ann..

[59] DePristo M.A., Banks E., Poplin R., Garimella K.V., Maguire J.R., Hartl C., Philippakis A.A., Angel G.D., Rivas M.A., Hanna M. (2011). A framework for variation discovery and genotyping using next-generation DNA sequencing data. Nat. Genet..

[60] Kosambi D.D. (1943). The estimation of map distance from recombination values. Ann. Eugen..

