# Supplementary material for: Predominantly Independent Genetic Control Between Growth and Visceral White Nodules Disease Resistance Revealed by High-Density Linkage Map and QTL Mapping in Larimichthys crocea
Source: Int J Mol Sci. 2026 Mar 10;27(6):2531. doi: 10.3390/ijms27062531 (PMC13026201; doi:10.3390/ijms27062531)
Supplement: Supplementary file 1 [file ijms-27-02531-s001.zip › Supplementary Table 1.pdf]

Table S1

Descriptive statistics and normality test of phenotype traits.

| | Mean | Min | Max | Median | sd | CV (%) | skew | kurtosis |
|---|---|---|---|---|---|---|---|---|
| AL | 146.90 | 90.26 | 212.2 | 147.41 | 21.89 | 14.9 | 0.02 | 0.01 |
| BL | 121.11 | 64.14 | 180.5 | 121.29 | 20.55 | 17.0 | -0.04 | 0.13 |
| CH | 37.30 | 22.2 | 56.41 | 37.04 | 6.51 | 17.5 | 0.02 | -0.22 |
| CL | 33.64 | 20.88 | 44.2 | 33.90 | 4.35 | 12.9 | -0.27 | 0.23 |
| QL | 49.28 | 19.71 | 80.4 | 48.93 | 10.56 | 21.4 | -0.12 | 0.28 |
| TH | 8.85 | 5.33 | 13.57 | 8.95 | 1.65 | 18.6 | 0.18 | -0.45 |
| TL | 25.46 | 10.54 | 40.04 | 25.15 | 5.21 | 20.5 | 0.19 | 0.16 |
| WL | 38.98 | 23.95 | 57.22 | 39.02 | 6.12 | 15.7 | 0.07 | 0.27 |
| Wt | 33.29 | 7.4 | 74 | 30.8 | 14.71 | 44.2 | 0.49 | -0.48 |
| AT | 54.89 | 25 | 102.1 | 58.6 | 16.99 | 31.0 | -0.30 | -0.80 |
| PPLL | 0.16 | 0.01 | 0.45 | 0.13 | 0.11 | 68.8 | 0.97 | 0.28 |
| PPSL | 0.08 | 0.00008 | 0.33 | 0.03 | 0.09 | 112.5 | 1.11 | 0.16 |
