# Supplementary material for: Predominantly Independent Genetic Control Between Growth and Visceral White Nodules Disease Resistance Revealed by High-Density Linkage Map and QTL Mapping in Larimichthys crocea
Source: Int J Mol Sci. 2026 Mar 10;27(6):2531. doi: 10.3390/ijms27062531 (PMC13026201; doi:10.3390/ijms27062531)
Supplement: Supplementary file 1 [file ijms-27-02531-s001.zip › Supplementary Table 2.pdf]

Table S2 Confidence intervals for key QTLs.

	LG	Total (cM)	Low (cM)	Peak (cM)	Up (cM)	Lod	Length (cM)
AL	6	56.350	42.012	42.023	42.345	5.745	0.333
			52.345	54	56.35	3.738	4.005
	16	46.002	0	2.000	2.334	3.841	2.334
			13.001	13.009	13.334	5.134	0.333
			27.668	28	28.668	3.496	1
	23	61.674	35.334	35.668	35.676	4.259	0.334
			53.668	54.002	54.013	3.019	0.334
BL	6	56.350	42.012	42.023	42.345	6.382	0.333
			52.345	55	56.35	4.293	4.005
	16	46.002	0	2.000	2.334	3.948	2.334
			13.001	13.009	13.334	5.030	0.333
			25.668	26.012	27.001	3.833	1.333
	23	61.674	35.334	35.668	35.676	3.525	0.334
CH	6	56.350	41.012	42.023	42.345	6.984	1.333
			52.345	54	56.35	4.581	4.005
	16	46.002	0	2.000	2.334	3.820	2.334
			13.001	13.009	13.334	5.044	0.333
			25.668	26.012	27.001	3.203	1.333
	23	61.674	35.334	35.668	35.676	3.439	0.334
Wt	6	56.350	39.012	39.355	39.679	6.593	0.667
			49.012	49.357	49.679	5.244	0.667
	16	46.002	0	2.000	2.334	3.768	2.334
			13.001	13.009	13.334	4.274	0.333
	23	61.674	35.334	35.668	35.676	3.369	0.334
AT	22	55.002	5	5.335	5.667	3.566	0.667
PPLL	22	55.002	51.335	51.668	51.680	3.050	0.333
PPSL	22	55.002	48.668	49.001	49.012	3.089	0.333
