# Supplementary figures and images for: Predominantly Independent Genetic Control Between Growth and Visceral White Nodules Disease Resistance Revealed by High-Density Linkage Map and QTL Mapping in Larimichthys crocea
Source: Int J Mol Sci. 2026 Mar 10;27(6):2531. doi: 10.3390/ijms27062531 (PMC13026201; doi:10.3390/ijms27062531)

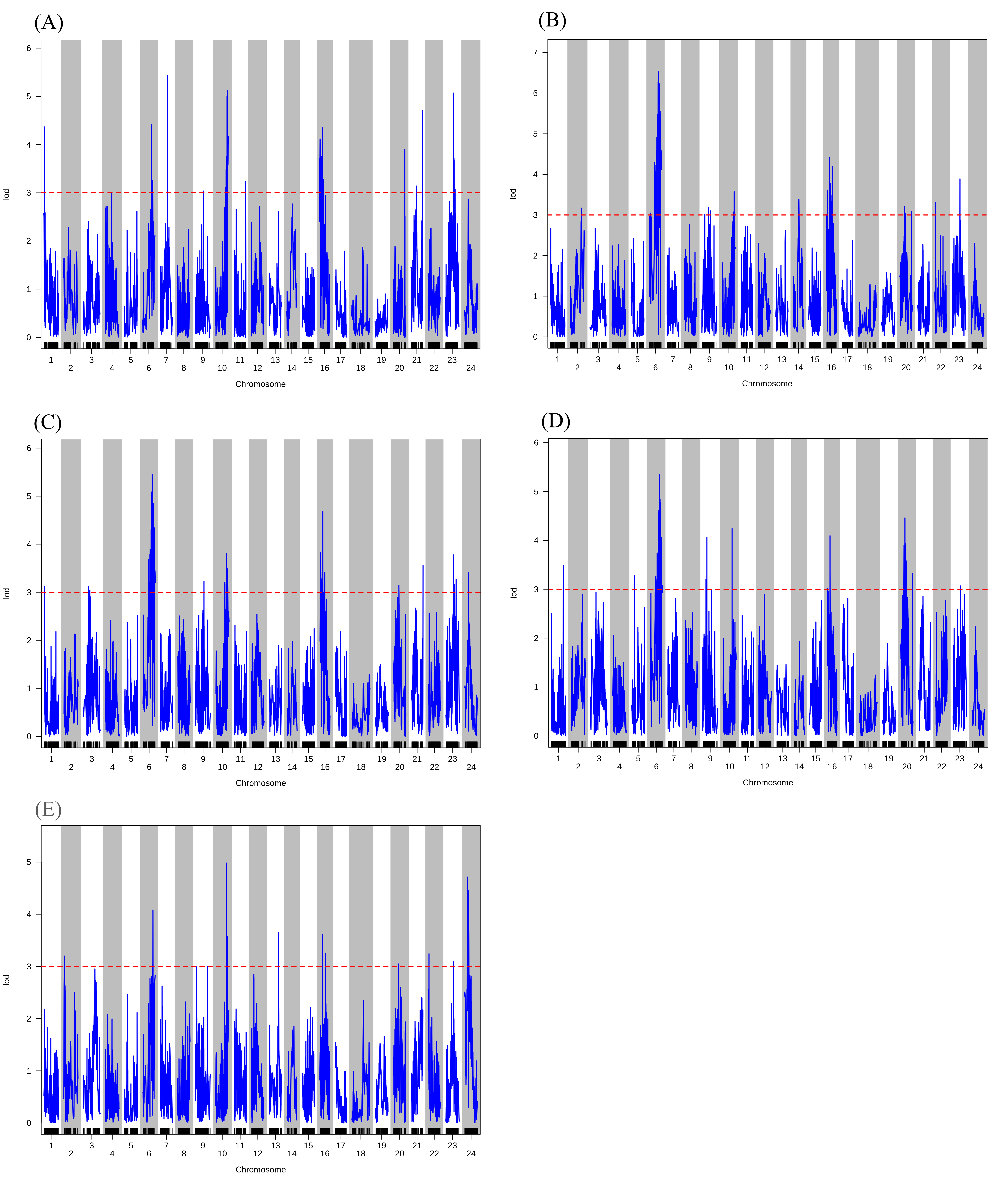

Supplement: Supplementary file 1 [file ijms-27-02531-s001.zip › Supplementary Figure S1.png]
